# Geospatial Analysis of Opioid-Related Outcomes Across Riverside County ZIP Codes and Associations With Social Determinants of Health

**DOI:** 10.7759/cureus.114997

**Published:** 2026-08-22

**Authors:** Brandon Ta, Omeka Ikegbu, Gerard Limerick, Paul Christo, Anita Gupta

**Affiliations:** 1 College of Natural and Agricultural Sciences, University of California, Riverside, Riverside, USA; 2 School of Medicine, University of California, Riverside, Riverside, USA; 3 Physical Medicine and Rehabilitation, Johns Hopkins University School of Medicine, Baltimore, USA; 4 Anesthesiology and Critical Care, Johns Hopkins University School of Medicine, Baltimore, USA

**Keywords:** geospatial mapping, opioid overdose, riverside county, social determinants of health, zip code disparities

## Abstract

Background

This study evaluated ZIP-code-level variation in opioid-related emergency department visits, hospitalizations, and overdose deaths across Riverside County, California, and examined associations with community-level social determinants of health (SDOH), operationalized using Healthy Places Index (HPI) domains and indicators. Identifying ZIP codes with elevated opioid-related outcome severity may help prioritize interventions such as naloxone distribution, mobile outreach, treatment linkage, and community education.

Methods

Publicly available data from the California Department of Public Health was analyzed to capture opioid-related deaths, hospitalizations, and emergency department visits in Riverside County. Datasets were cleaned and integrated using R (R Foundation for Statistical Computing, Vienna, Austria). A normalized severity score (NSS) was calculated for each ZIP code by combining mortality, hospitalizations, and ED visits into a composite min-maxed score from 0-1. Geographic variation was visualized using an interactive choropleth map developed with the Leaflet package. Multivariable linear regression was used to evaluate associations between the NSS and community-level HPI domains and indicators, including measures of economic conditions, housing, transportation, employment, and homeownership.

Results

NSS values varied substantially across Riverside County ZIP codes. In multivariable analyses, lower Economic and Transportation HPI domain scores and lower employment and homeownership indicators were associated with higher NSS values.

Conclusions

Higher NSS values were associated with lower Economic and Transportation HPI domain scores, as well as lower employment and homeownership indicators. Integrating fatal and non-fatal outcomes into the NSS provides a broader descriptive measure of community-level opioid burden than examining mortality alone. These geospatial findings may help guide location-specific community outreach and preventive interventions in ZIP codes with higher NSS values. Additionally, the geographic variation observed across Riverside County ZIP codes is broadly consistent with national evidence of spatial variation in opioid-related morbidity and mortality.

## Introduction

Opioid-related deaths in the United States remain a major public health concern. In 2023, nearly 80,000 deaths involved opioids nationwide [1]. California recorded 11,359 drug-related overdose deaths during the same year [2]. The continued evolution of the crisis has been driven in part by the increasing prevalence of illicitly manufactured synthetic opioids such as fentanyl [3].

The opioid crisis has evolved through successive phases involving prescription opioids, heroin, and illicitly manufactured synthetic opioids. More recently, deaths involving multiple substances, including synthetic opioids and psychostimulants, have become increasingly prominent [3]. These changes demonstrate that the epidemic is a complex and evolving phenomenon shaped by both drug-market conditions and social determinants of health.

The evolution of the opioid epidemic demonstrates that opioid-related burden cannot be explained by changes in prescribing or drug supply alone. Dasgupta et al. argued that the crisis is also shaped by economic and social upheaval, physical and psychological trauma, concentrated disadvantage, social isolation, and hopelessness [4]. The National Academies of Sciences, Engineering, and Medicine similarly framed the epidemic as requiring efforts to reduce opioid-related adverse outcomes while preserving appropriate and effective pain management [5]. These perspectives support examining the structural and community-level conditions that may influence local patterns of opioid-related morbidity and mortality.

Although national and state estimates demonstrate the magnitude of the epidemic, they can obscure substantial geographic variation among counties and local communities. Monnat found that county-level drug-related mortality was higher in areas characterized by greater economic and family distress [6]. Rural-urban differences may also reflect variation in prescription opioid availability, population out-migration, social network structure, and economic stress [7]. At the ZIP-code level, Pear et al. found that prescription opioid overdose-related hospital discharges were concentrated in economically disadvantaged communities across levels of urbanicity, while the socioeconomic correlates of heroin overdose varied between urban and rural areas [8]. Geographic disparities also extend to access to treatment. Haffajee et al. identified counties with persistently high opioid overdose mortality but limited capacity to provide medications for opioid use disorder [9].

These findings support examining combined fatal and nonfatal opioid-related outcomes at smaller geographic scales. Kolak et al. demonstrated that multidimensional social determinants of health can be quantified across census tracts, revealing neighborhood-level patterns that may be obscured by larger geographic units [10]. In Riverside County, Rey et al. identified significant space-time clustering of fatal opioid overdoses and found that overdose locations were disproportionately situated in neighborhoods with lower incomes, lower housing values, and lower educational attainment [11].

Existing spatial studies have frequently evaluated individual dimensions of opioid burden. For example, Pear et al. assessed opioid-related hospital discharges, whereas Rey et al. focused specifically on fatal overdoses [8,11]. These studies leave an opportunity to integrate emergency department visits, hospitalizations, and deaths into a single ZIP-code-level measure. Analyses limited to overdose deaths may not fully characterize communities experiencing substantial numbers of opioid-related ED visits and hospitalizations. Furthermore, the relationship between community-level social determinants of health, as measured using Healthy Places Index (HPI) domains and indicators, and a composite measure incorporating fatal and nonfatal opioid outcomes remains insufficiently characterized at the subcounty level.

To address these gaps, this study had three objectives: (1) to develop a ZIP-code-level Normalized Severity Score (NSS) integrating opioid-related emergency department visits, hospitalizations, and overdose deaths; (2) to map the geographic distribution of opioid severity across Riverside County to identify areas with elevated opioid-related burden; and (3) to evaluate associations between opioid severity and community-level social determinants of health using HPI domains and indicators. The primary objective was to characterize geographic variation in overall opioid burden at the ZIP-code level, while the secondary objective was to examine the social and structural factors associated with this variation. 

This ecological study was designed to provide descriptive and associational analyses of ZIP-code-level opioid burden rather than to develop a predictive model or establish causal relationships.

## Materials and methods

This study utilized three publicly available data sources: the California Department of Public Health (CDPH) California Overdose Surveillance Dashboard, the HPI database, and Data Commons population estimates [12-14]. For this study, social determinants of health (SDOH) were operationalized as the community-level socioeconomic and structural conditions represented by the selected HPI domains and indicators. These measures characterize conditions at the ZIP-code level and should not be interpreted as individual-level characteristics. The geographic scope of the study included ZIP codes in Riverside County, California. All analyses used five-digit ZIP-code identifiers; ZIP+4 codes were not used. Where census-derived population estimates or geographic boundaries were required, five-digit ZIP-code identifiers were matched to corresponding ZIP Code Tabulation Areas (ZCTAs). For this study, geographic disparities refer to differences in opioid-related outcomes across Riverside County ZIP codes, quantified using outcome-specific incidence rate ratios (IRRs) and the NSS described below. Opioid-related outcome data from the CDPH California Overdose Surveillance Dashboard were limited to the year 2023 [12]. Opioid-related outcomes were defined according to the outcome-specific opioid classifications used by the CDPH California Overdose Surveillance Dashboard. For overdose deaths, opioid involvement was identified using ICD-10 codes T40.0-T40.4 and T40.6. For hospitalizations and ED visits, the applicable ICD-10-CM opioid category included F11.0 and T40.0-T40.4 and T40.6. Psychostimulant-only events were not classified as opioid-related outcomes; however, polysubstance events involving both an opioid and a psychostimulant could be included when an opioid code was also present [12]. 

For this study, opioid-related morbidity was operationalized as opioid-related ED visits and hospitalizations, whereas opioid-related mortality was operationalized as opioid-related overdose deaths. ZIP-code-level population estimates for 2023 were obtained through Data Commons using the Count_Person statistical variable from the U.S. Census Bureau American Community Survey (ACS) 5-Year Survey data facet and were matched to the corresponding ZIP codes for estimation of opioid-related outcome counts. HPI predictor variables were drawn from the most recent available year for each indicator because consistent ZIP-code-level reporting across a single common year was not available. Consequently, HPI predictors ranged from 2017 to 2021 and preceded the 2023 opioid outcome data. The following HPI predictors and their respective data years were used at the ZIP-code level: Above Poverty (2019); Economic Domain (2021); Healthcare Access Domain (2021); Education Domain (2021); Homeownership (2019); Housing Domain (2021); Neighborhood Domain (2021); Insured Adults (2019); Low-income Homeowner Severe Housing Cost Burden (2017); Social Domain (2021); Transportation Domain (2021); Per Capita Income (2019); Employed (2019); and Bachelor’s Education or Higher (2019) [13].

The HPI domain scores represent composite measures of their constituent community-level indicators, with higher scores indicating healthier or more advantaged community conditions. The Economic Domain includes employment, per capita income, and above-poverty measures; the Education Domain includes bachelor’s educational attainment, high school enrollment, and preschool enrollment; the Social Domain includes voting participation and census response; the Transportation Domain includes automobile access and active commuting; the Healthcare Access Domain measures adult health insurance coverage; the Neighborhood Domain includes park access, tree canopy, and retail density; and the Housing Domain includes homeownership, housing habitability, uncrowded housing, and housing cost burden among low-income renters and homeowners. Individual HPI indicators included in this analysis were defined as follows: Above Poverty was the percentage of the population with income exceeding 200% of the federal poverty level; Employed was the percentage of people aged 20-64 who were employed; Per Capita Income represented mean income per person during the preceding 12 months; Bachelor’s Education or Higher was the percentage of people over age 25 with at least a bachelor’s degree; Insured Adults was the percentage of adults aged 18-64 with health insurance; Homeownership was the percentage of occupied housing units occupied by their owners; and Low-Income Homeowner Severe Housing Cost Burden was the percentage of low-income homeowner households, defined as households with income at or below 80% of the HUD area median family income, spending more than 50% of household income on housing costs [13].

All analyses were conducted in R (version 4.4.2; R Foundation for Statistical Computing, Vienna, Austria) using RStudio (Posit Software, PBC, Boston, MA, USA).

**Figure 1:**
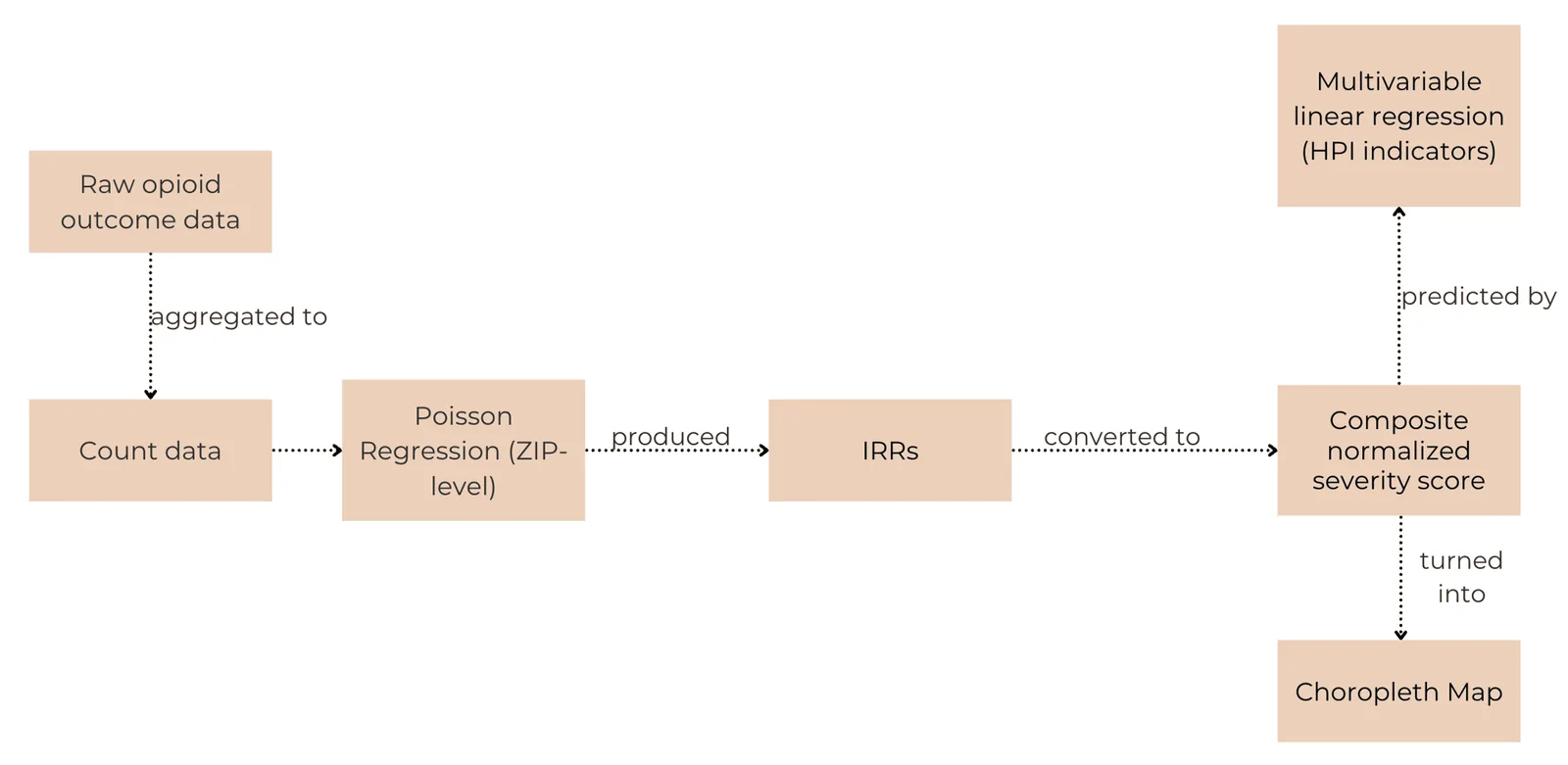
This study’s analytical pipeline from raw opioid outcome data to Normalized Severity Score (NSS), spatial visualization, and multivariable linear regression. HPI: Healthy Places Index [13]; IRR: incidence rate ratio

Beginning from the raw data obtained from the CDPH California Opioid Overdose Dashboard, the general analytical pipeline of this study is shown in Figure 1 below.

Each opioid outcome followed the same procedure to prepare for Poisson regression. Because the CDPH California Opioid Overdose Dashboard did not include counts, but age-adjusted rates per 100,000 persons per year, approximate counts for each outcome were calculated as the reported age-adjusted rate multiplied by the corresponding ZIP-code population and divided by 100,000. ZIP codes were treated as categorical variables, and the ZIP code with the closest outcome count to the median count was assigned as the reference level for the Poisson regression models. The outcome-specific reference ZIP codes were 92557 for emergency department visits, 92505 for hospitalizations, and 92882 for overdose deaths.

A Poisson regression model was run for each opioid outcome (deaths, hospitalizations, and emergency department visits) using estimated counts derived from rates taken from CDPH. This approach was well-suited for non-negative, integer-valued data and allowed for the calculation of IRRs for each specific outcome to be assigned to every ZIP code. This enabled ZIP-code-level geographic comparison of opioid-related emergency department visits, hospitalizations, and overdose deaths. 

After running separate Poisson regression models for each outcome, each ZIP code was assigned an incidence rate ratio (IRR) for each respective outcome. To reflect the clinical severity of each event type, a raw severity score was calculated by assigning greater weight to more severe outcomes: overdose deaths were weighted as 3, hospitalizations as 2, and ED visits as 1. The raw severity score for each ZIP code was computed as a weighted sum of its three IRRs.

\[ Severity_{ZIP} = 1 \cdot IRR_{ED,ZIP} + 2 \cdot IRR_{Hosp,ZIP} + 3 \cdot IRR_{Death,ZIP} \]

A sensitivity analysis evaluated the robustness of ZIP-code severity rankings to alternative weighting schemes. The primary 1:2:3 weighting scheme was compared with an equal-weight 1:1:1 scheme and a mortality-emphasized 1:2:4 scheme. For each alternative scheme, severity scores were recalculated and normalized using the same min-max procedure as the primary NSS. Spearman rank correlations were then calculated between the primary NSS and each alternative NSS.

The resulting severity scores were transformed to the NSS using min-max normalization:

\[ NSS_{ZIP} = \frac{Severity_{ZIP} - \min(Severity)}{\max(Severity) - \min(Severity)} \]

The minimum and maximum severity values were calculated across all included Riverside County ZIP codes, producing NSS values ranging from 0 to 1. In this study, opioid burden refers to the combined burden of opioid-related ED visits, hospitalizations, and overdose deaths as represented by the NSS; higher NSS values indicate greater relative opioid burden among the included ZIP codes. Opioid-related outcome severity refers specifically to this NSS-based measure of combined fatal and nonfatal opioid outcomes and should not be interpreted as the clinical severity of individual overdose events.

The NSS was intended to reflect the differing clinical severity of opioid-related outcomes while preserving information from both fatal and non-fatal outcomes. ED visits were weighted lowest because they represent acute opioid-related encounters that may not require inpatient care, while hospitalizations were weighted more heavily because they likely represent more severe morbidity requiring higher levels of medical intervention. Fatal overdoses were weighted highest because they represent the most severe outcome. This approach allows the severity score to capture a broader spectrum of opioid burden than mortality alone. However, because the weighting scheme is author-defined, it should be interpreted as a pragmatic severity measure rather than a validated clinical index.

An interactive choropleth map was created using each ZIP code’s normalized severity score to descriptively visualize geographic variation in opioid burden across Riverside County. The map was not used as a formal statistical test of spatial clustering or hotspot significance; methods such as Moran’s I, Getis-Ord Gi*, or kernel density estimation were not performed.

For each multivariable regression model, complete-case analysis was used; ZIP codes with missing values for the NSS or any candidate predictor included in the corresponding full model were excluded prior to standardization and model fitting. Before creating multivariable linear regression models, all HPI predictors were standardized using z-score normalization (where the mean = 0 and the standard deviation = 1) to mitigate scale bias and to effectively compare the predictor’s effect on opioid outcomes. Initial models included all candidate indicators and domains mentioned above. Variable selection was performed using bidirectional stepwise Akaike Information Criterion (AIC) optimization implemented with MASS::stepAIC(), allowing both addition and removal of candidate predictors. Multicollinearity was assessed using the Variance Inflation Factor (VIF). Redundancy among predictors was further addressed through the model variable selection process. All retained predictors had VIF values below 6, indicating no evidence of severe multicollinearity. 

To identify ZIP-level predictors of opioid-related outcomes, two multivariable linear regression models were constructed using NSS as the dependent variable. The first multivariable regression model included HPI domains as independent variables to evaluate their correlation with opioid severity scores. The second multivariable regression model included HPI indicators as independent variables to test their correlation with the severity scores. These predictors included: economic domain, social domain, healthcare access, education, housing, transportation, and others listed above. 

The final models were used to identify the HPI domains and indicators independently associated with the NSS. All data cleaning, transformation, modeling, and visualization were performed in R (version 4.4.2) using RStudio. Key packages used in RStudio include: dplyr, tidyverse for data wrangling; ggplot2 for static plotting; MASS, car for regression modeling and diagnostics; sf, tigris, leaflet for geospatial visualization; RColorBrewer, htmltools for interactive map styling and rendering; gt for table creation.

The complete analysis workflow, including Poisson regression, multivariable linear regression, map development, and data visualization, was executed entirely in RStudio.

## Results

During 2023, Riverside County had an estimated 3,509 total opioid-related incidents. Included incidents represented one of three possible outcomes: opioid-related ED visits, hospitalization, and overdose deaths. There were an estimated 2,538 opioid-related ED visits, 375 hospitalizations, and 596 overdose deaths. Table 1 summarizes these overall statistics.

**Table 1:** Distribution of Opioid-Related Outcomes Across Riverside County ZIP Codes in 2023 Counts estimated as age-adjusted rate × ZIP-code population / 100,000.

Outcome	Median	IQR	Range
ED visits	21	38	0-264
Hospitalizations	4	7	0-39
Overdose deaths	9	10	1-35

Poisson regression models based on these estimated counts yielded raw coefficients for opioid-related ED visits, hospitalizations, and overdose deaths across all Riverside County ZIP codes. These coefficients were exponentiated to become IRRs for interpretability. IRRs for opioid-related ED visits ranged from <0.01 to 30.07. IRRs for opioid-related hospitalizations ranged from <0.01 to 12.644. IRRs for opioid overdose deaths ranged from 0.339 to 7.043. IRRs allow for ZIP-level comparison because they quantify the relative rate of opioid health outcomes across each ZIP code. Each IRR was expressed relative to an outcome-specific reference ZIP code, selected as the ZIP code whose estimated count was closest to the median estimated count for that outcome. The reference ZIP code therefore had an IRR of 1. Summary statistics of IRRs across ZIP codes are presented in Table 2, highlighting substantial variability across geographic areas. Extreme IRR values, particularly for emergency department visits, should be interpreted cautiously because estimates for ZIP codes with relatively small populations or low estimated counts may be less stable.

**Table 2:** IRR Summary by Outcome Riverside County ZIP codes (2023) IRR: incidence rate ratio

Outcome	Min	Q1	Median	Mean	Q3	Max
ED visits	<0.01	1.219	1.881	3.353	2.974	30.07
Hospitalizations	<0.01	0.588	1.436	2.096	2.36	12.644
Overdose deaths	0.339	1.105	2.13	2.524	3.55	7.043

**Figure 2:**
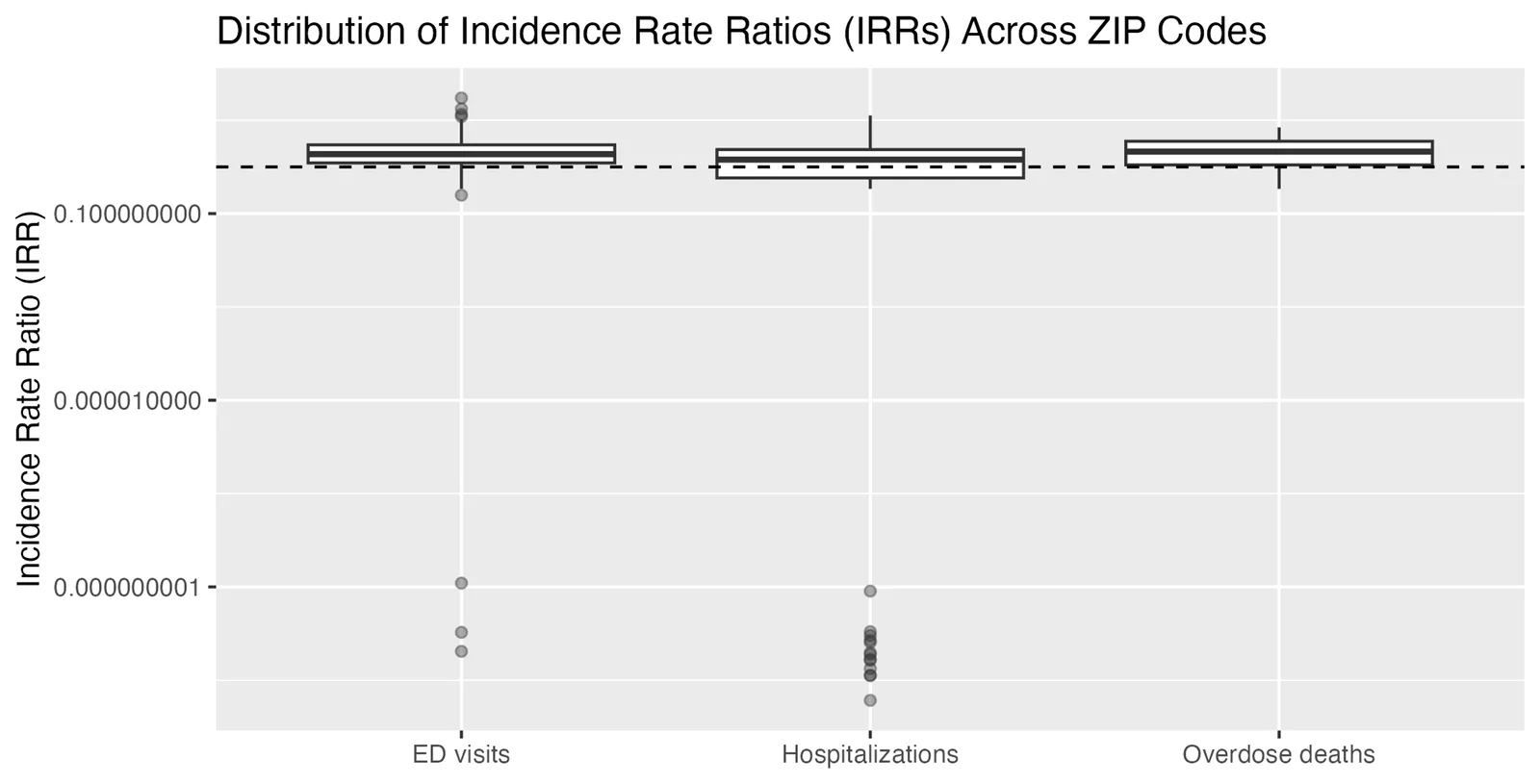
Boxplots display the distribution of IRRs for ED visits, hospitalizations, and overdose deaths. The dashed line represents the reference IRR of 1.

Importantly, ED visit rates due to opioid-related incidents varied drastically across ZIP codes. Most ZIP codes had rates approximately one to three times that of the outcome-specific reference ZIP code. This finding suggests that ZIP codes with elevated burden drive a disproportionate share of ED encounters. Figure 2 displays the distribution of these IRRs.

Across all three outcomes, most ZIP-code-level IRRs fell within a relatively compact range, although meaningful dispersion and outcome-specific variability were observed, particularly for emergency department visits. Meanwhile, the distribution of fatal overdoses was more tightly concentrated, indicating lower variability across ZIP codes compared to ED visits and hospitalizations. These patterns support the generation of a raw composite severity score based on IRRs and subsequent multivariable modeling to identify structural predictors of opioid burden.

**Figure 3:**
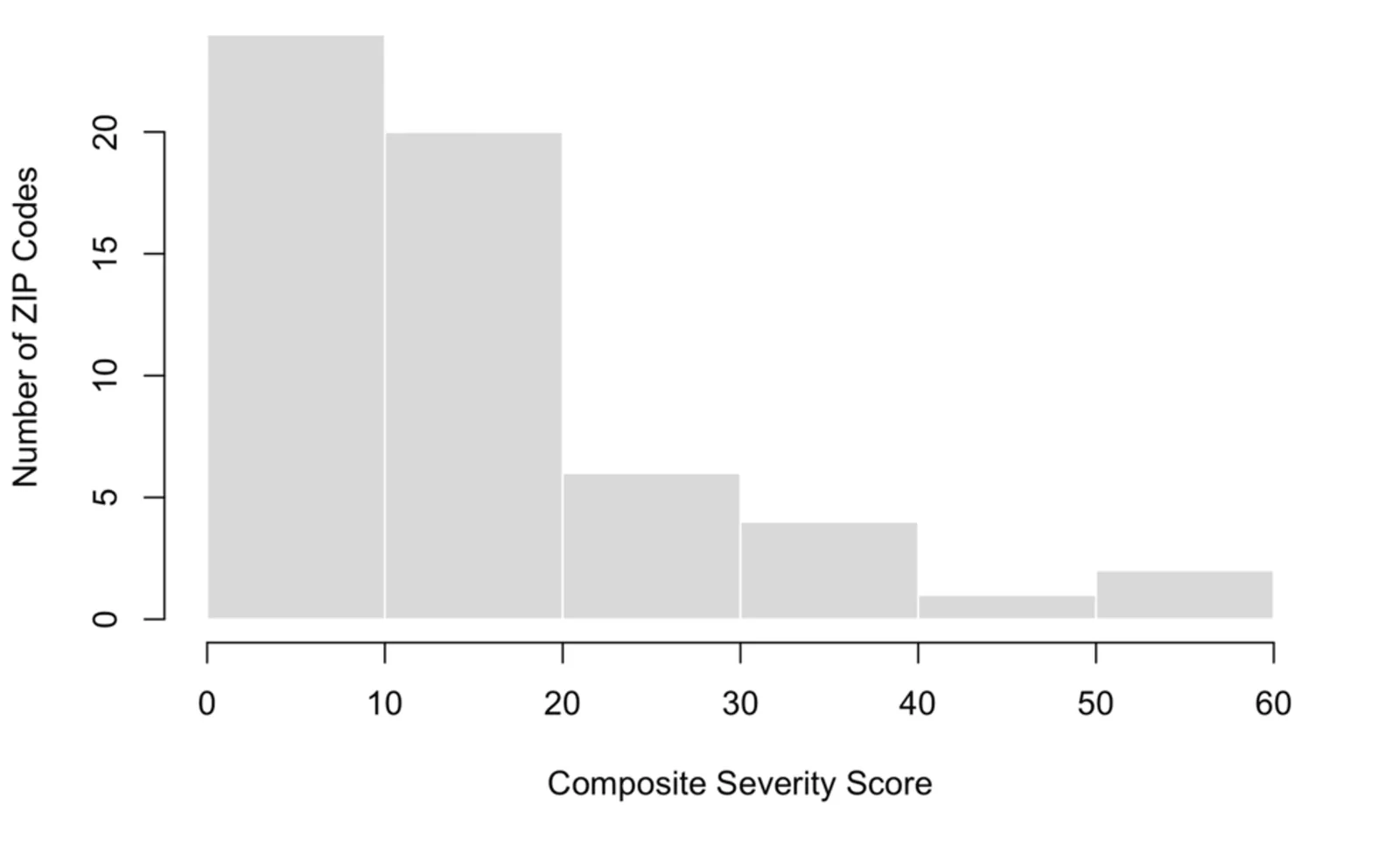
Distribution of raw composite opioid severity scores across Riverside County ZIP codes.

Individual outcome IRRs were combined to form the raw composite severity scores for each Riverside County ZIP code. Raw composite severity scores were calculated for each Riverside ZIP code by weighting outcomes by severity: ED visits = 1, hospitalizations = 2, and overdose deaths = 3. Figure 3 displays the distribution of these raw composite scores.

As illustrated in Figure 3, there is a clear right-skewed distribution of raw composite opioid severity scores. While most ZIP codes have low to moderate severity scores, there are some ZIP codes that are disproportionately higher in severity scores. This pattern indicates that opioid-related burden is not evenly distributed across the county. Instead, it is concentrated within a limited number of ZIP codes. The presence of a long right tail reflects the ZIP codes experiencing disproportionately high combined burdens of emergency department visits, hospitalizations, and overdose deaths. Sensitivity analyses demonstrated that ZIP-code severity rankings were highly consistent across alternative weighting schemes. The primary 1:2:3 weighting scheme was strongly correlated with both the equal-weight 1:1:1 scheme (Spearman ρ = 0.977) and the mortality-emphasized 1:2:4 scheme (Spearman ρ = 0.992).

**Figure 4:**
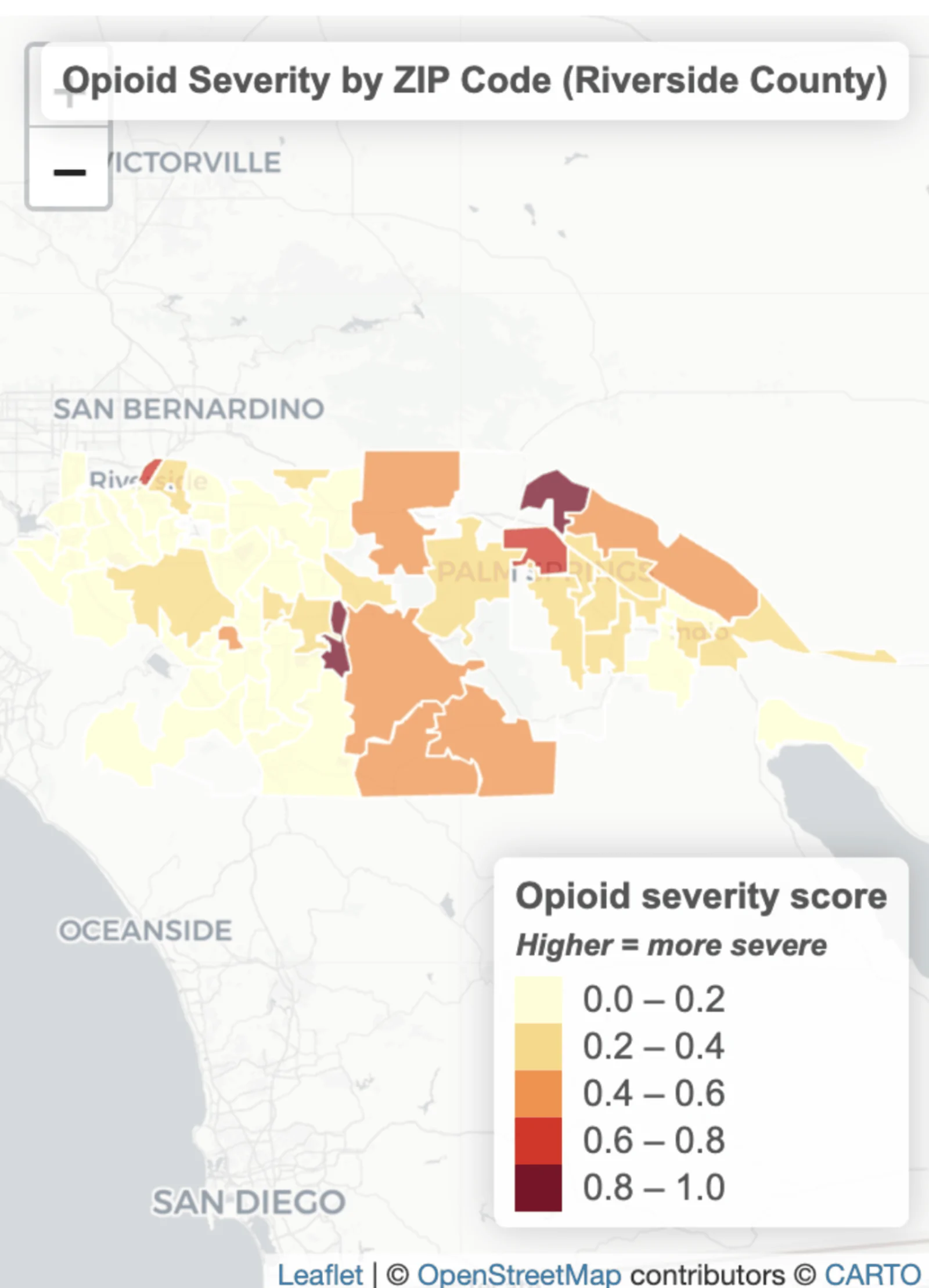
Choropleth map of Normalized Severity Scores (NSS) across Riverside County ZIP codes, illustrating spatial heterogeneity in opioid-related health outcomes. Original visualization generated by the authors using publicly available data from the California Department of Public Health Overdose Surveillance Dashboard and the Healthy Places Index [12,13]. Basemap attribution: © OpenStreetMap contributors © CARTO. This figure was not reproduced or adapted from a previously published figure.

While Figure 3 demonstrates the overall distribution and heterogeneity of raw composite opioid severity scores across ZIP codes, it does not convey their spatial arrangement. Using the NSS, a choropleth map was generated to highlight the geographic distribution of the severity scores across Riverside County ZIP codes, represented by Figure 4.

The choropleth demonstrates substantial geographic disparity among ZIP codes. Higher severity scores were predominantly observed in the south and southeastern region of Riverside County. In contrast, lower severity scores were predominantly observed in the western region of the county. The observed spatial pattern suggests that regions with higher severity scores may suffer from localized structural or socioeconomic factors. These specific regions may benefit from targeted public health interventions.

This spatial pattern is important because Riverside County contains communities with substantially different levels of SDOH. A county-level estimate would treat these areas as a single unit and obscure the localized concentration of opioid-related burden. By identifying ZIP codes with higher normalized severity scores, the map provides a practical framework for prioritizing outreach, naloxone distribution, treatment linkage, and further local investigation. Importantly, the map should be interpreted as a tool for identifying areas of elevated burden rather than as evidence that any individual resident within those ZIP codes is at higher risk.

While the choropleth map (Figure 4) illustrates the geographic distribution of NSS across Riverside County ZIP codes, it does not identify the factors associated with this disparity. To examine potential predictors of opioid severity, multivariable linear regression models were constructed using HPI domains and indicators as explanatory variables. The HPI is a composite measure of community-level socioeconomic and environmental conditions, calculated from multiple indicators across domains including economic, education, housing, transportation, and healthcare access. Each ZIP code is assigned a percentile score (0-100), with higher values indicating healthier community conditions.

**Figure 5:**
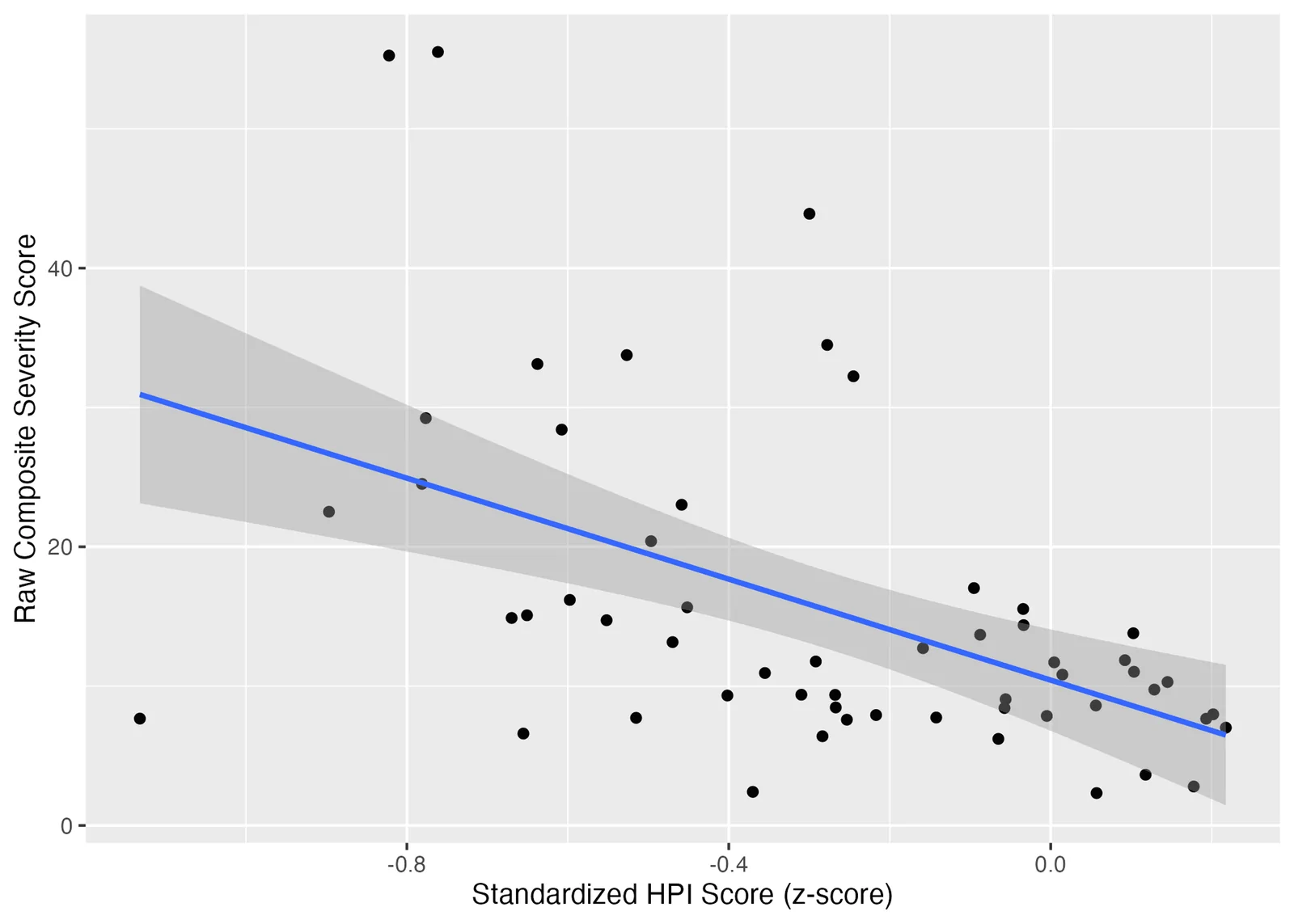
Scatterplot illustrating the relationship between standardized Healthy Places Index (HPI) score and raw composite opioid severity score.

Prior to generating multivariable regression models, a scatterplot was generated to explore the bivariate relationship between standardized HPI score and raw composite severity score, as shown in Figure 5.

As shown in Figure 5, there appears to be a modest negative association between HPI score and severity score. Higher standardized HPI scores generally corresponded to lower opioid severity scores. While the relationship is weak and contains substantial variability, the observed trend suggests that ZIP-level health conditions may be associated with opioid-related outcomes. This exploratory finding supported further investigation using multivariable regression modeling to account for potential confounding factors and to assess the independent contributions of specific HPI components.

Following this exploratory bivariate analysis, two multivariable linear regression models were fitted using the NSS as the dependent variable. The first model included HPI domains as predictors, while the second included individual HPI indicators. Results from the domain- and indicator-level models are presented in Tables 3, 4, respectively.

**Table 3:** Multivariable Linear Regression of Normalized Severity Scores (NSS) on Healthy Places Index (HPI) Domains

Characteristic	Beta	95% Confidence Interval	p-value
Housing domain	0.06	-0.01, 0.13	0.072
Economic domain	-0.12	-0.19, -0.06	<0.001
Transportation domain	-0.09	-0.15, -0.03	0.002
R^2^	0.45	-	-
Adjusted R^2^	0.418	-	-

**Table 4:** Multivariable Linear Regression of Normalized Severity Scores (NSS) on Healthy Places Index (HPI) Indicators

Characteristic	Beta	95% Confidence Interval	p-value
Homeownership	-0.06	-0.11, -0.02	0.006
Employed	-0.14	-0.18, -0.09	<0.001
R^2^	0.486	-	-
Adjusted R^2^	0.467	-	-

In the multivariable linear regression analyses evaluating HPI domains as predictors of NSS (Table 3), the economic and transportation domains emerged as the most robust independent predictors of opioid severity across Riverside County ZIP codes. Domains related to healthcare access, education, neighborhood environment and social context were not retained in the final stepwise model. The exclusion of these domains suggests that their associations with opioid severity may be explained by shared variance with other structural domains, particularly economic conditions. These findings indicate that while multiple aspects of community context are related to opioid burden, the Economic and Transportation HPI domains represent the main domain-level factors independently associated with opioid severity in this analysis.

While the domain-level analysis in Table 3 reveals broad factors related to opioid severity, it does not go into detail about underlying characteristics within these domains that drive these associations. To further investigate, another multivariable linear regression was constructed using HPI indicators as predictors, with results presented in Table 4.

In the indicator-level multivariable regression analysis, indicators representing economic stability, housing conditions, education, income and healthcare access were evaluated. Of these indicators, homeownership and employment remained independently associated with severity score in the final stepwise model. The exclusion of other indicators suggests that their association with opioid burden may be explained by shared variance with employment and homeownership. These findings further support the underlying domain-level associations observed in Table 3. The final domain-level model explained 45.0% of the variance in NSS (adjusted R² = 0.418), while the final indicator-level model explained 48.6% of the variance (adjusted R² = 0.467). Thus, substantial variation in ZIP-code-level opioid severity remained unexplained by the measured HPI predictors.

Taken together, the domain- and indicator-level analyses demonstrate that economic and transportation conditions were negatively correlated with opioid outcome severity across Riverside County ZIP codes, while the housing domain showed a non-significant positive association. At the indicator level, higher employment and homeownership rates were both significantly negatively correlated with opioid severity, suggesting that greater economic stability is associated with lower opioid burden.

## Discussion

This study demonstrates that opioid-related burden is disproportionately distributed across ZIP codes in Riverside County. Using the NSS that integrated emergency department visits, hospitalizations, and overdose deaths, substantial geographic heterogeneity in opioid burden was identified. Multivariable linear regression analysis identified the economic and transportation domains as significant domain-level correlates of opioid severity, while the housing domain demonstrated a nonsignificant positive association. The weak association between the healthcare access domain and opioid severity may reflect covariance with other social determinant domains, including economic conditions. It may also reflect limitations in how healthcare access is captured by ZIP code-level HPI indicators, which may not fully account for the opioid-related care and harm reduction resources that are available or accessible in each ZIP code. 

In the multivariable linear regression model, the housing domain had a positive correlation coefficient. This may be influenced by collinearity with the other social determinant domains. However, this association was nonsignificant (p = 0.072). In contrast, indicator-level analyses showed a negative association between homeownership and opioid severity, highlighting potential heterogeneity within housing-related determinants. The other two domains retained in the multivariable linear regression model, transportation and economic domains, were significant negative correlates. These findings suggest that ZIP codes experiencing transportation-related disadvantages and socioeconomic barriers may have higher opioid-related burden, potentially reflecting broader barriers to prevention, treatment, and emergency response resources. Although these associations do not establish causal effects or individual-level mechanisms, the observed ZIP-code-level patterns may help inform further investigation, outreach, and resource planning.

Further multivariable linear regression analysis at the indicator-level suggested that employment and homeownership may underscore broader domain-level associations. Lower scores in homeownership and employment were associated with higher opioid severity, reinforcing the results found in the domain-level multivariable analysis. Lower homeownership scores may reflect broader housing instability and socioeconomic disadvantage within a ZIP code, which may help explain higher community-level opioid burden. However, because this analysis did not directly measure individual housing status or experiences of stigma, these mechanisms should be interpreted cautiously. Additionally, the negative association between employment and opioid severity may reflect broader community-level economic stability and transportation access, though individual employment status and commuting access cannot be inferred from these ZIP code-level data. 

These findings support the conclusion that social determinants of health are strong correlates of opioid severity in Riverside County. Given the county’s geographic and demographic diversity, ZIP codes span urban, suburban, and rural areas with distinct structural conditions that may be associated with opioid-related burden. Prior literature has indicated that economic instability, social isolation, limited healthcare access, and community-level trauma are associated with higher community-level risk for substance use disorder morbidity and overdose [4,15]. This perspective and the analysis of opioid outcomes in Riverside County support consideration of upstream SDOH when interpreting and responding to opioid-related burden.

Furthermore, the findings from the present analysis are consistent with prior spatial analyses of opioid outcomes in Riverside County. Rey et al. identified significant spatial and space-time clustering of fatal opioid overdoses, with higher burden concentrated in socioeconomically disadvantaged and structurally marginalized areas [11]. While their study focused exclusively on fatal overdoses and utilized space-time interaction methods, the present analysis extends this work by incorporating the non-fatal outcomes of emergency department visits and hospitalizations into a composite severity metric. Together, these findings are consistent with associations between SDOH and broader patterns of both fatal and non-fatal opioid-related burden across geographic areas.

These findings are also consistent with national evidence that opioid-related burden displays strong spatial and temporal clustering across the United States. A recent spatiotemporal analysis of substance use disorder mortality identified 27 significant clusters of elevated mortality across U.S. counties, with mortality rates nearly double within clusters compared to non-cluster regions [16]. These findings reinforce the importance of spatially targeted analyses, as opioid-related burden is not randomly distributed but instead reflects localized “micro-epidemics” shaped by underlying structural and socioeconomic conditions [16]. 

The geographic disparities observed in Riverside County are broadly consistent with national studies reporting spatial concentration of opioid- and substance-related burden. However, because the present analysis was limited to a single county, these similarities should not be interpreted as evidence that the Riverside County pattern is representative of national patterns. The presence of localized geographic disparities emphasizes the potential value of geographically targeted interventions.

This study highlights the importance of analyzing ZIP code-level data to understand the localized and uneven impact of the opioid crisis in Riverside County. However, there are several limitations to this study. Because this was an ecological analysis using aggregated ZIP-code-level data, the observed associations cannot establish causality and may not reflect individual-level risk, introducing the possibility of ecological fallacy. Additionally, spatial autocorrelation among neighboring ZIP codes was not explicitly modeled, and therefore the regression analyses may not fully account for geographic dependence between observations. Because the CDPH dashboard reported age-adjusted rates rather than raw event counts, counts used in the Poisson models were approximated from reported rates and ZIP-code population estimates. This back-calculation may introduce estimation error, and IRRs for ZIP codes with relatively small estimated counts or populations may be less stable and more susceptible to extreme values. Although opioid outcomes were measured using 2023 data, several HPI indicators were derived from 2017-2019 data and may not fully reflect current community-level conditions in Riverside County. Socioeconomic conditions may have shifted substantially during this period, particularly because of the COVID-19 pandemic, potentially introducing measurement error in the SDOH predictors. Overdose data may also be underreported, especially in unhoused populations, which could result in an underestimation of opioid-related burden in certain areas. Furthermore, unmeasured confounding variables, such as law enforcement activities, local prescribing practices, and the presence of opioid harm reduction services such as naloxone distribution practices were not accounted for in this analysis. Additionally, because this study was limited to Riverside County, the findings may not be generalizable to regions with substantially different demographic, geographic, or healthcare characteristics. Finally, the author-defined weighting scheme used to construct the NSS introduces subjectivity. However, sensitivity analyses using equal weighting and greater mortality weighting produced highly similar ZIP-code rankings, suggesting that the overall geographic ordering was robust to these alternative weighting assumptions.

These findings also demonstrate the practical value of ZIP-code-level analysis for public health research. In geographically large and diverse counties such as Riverside County, opioid-related burden may be diluted when examined only at the county level. ZIP-code analysis can help identify communities where prevention and response resources may be most urgently needed. This is especially relevant for interventions such as naloxone distribution, mobile outreach, linkage to medication-assisted treatment for opioid use disorder, and community education programs. Rather than applying uniform strategies across the entire county, public health agencies may benefit from using localized ZIP-level data to guide resource allocation.

In summary, this study demonstrates substantial geographic disparity in opioid-related incidents across ZIP codes in Riverside County. It identifies socioeconomic and structural conditions as key correlates of opioid severity. Through the integration of fatal and non-fatal outcomes into the NSS, this study’s analysis contributes an in-depth depiction of opioid burden across Riverside County. These results suggest that socioeconomic disadvantage and transportation-related barriers may be relevant considerations when developing and evaluating geographically targeted harm reduction strategies.

## Conclusions

This study demonstrates substantial variability in opioid-related burden across Riverside County ZIP codes in ways that reflect the underlying uneven distribution of social and structural disparities. By integrating opioid-related emergency department visits, hospitalizations, and overdose deaths into the NSS, this analysis captures fatal and non-fatal outcomes of opioid burden that may be missed when examining mortality alone. The use of ZIP code-level mapping further highlights localized geographic disparities that county-level analyses may obscure. Together, these findings emphasize the value of composite, community-level metrics for identifying areas of elevated opioid burden and guiding targeted public health strategies. Ultimately, these findings may help inform community-specific public health planning that considers local structural conditions when addressing opioid-related burden.
